# Reported Cases of End-Stage Kidney Disease — United States, 2000–2019

**DOI:** 10.15585/mmwr.mm7111a3

**Published:** 2022-03-18

**Authors:** Nilka Ríos Burrows, Alain Koyama, Meda E. Pavkov

**Affiliations:** 1 Division of Diabetes Translation, National Center for Chronic Disease Prevention and Health Promotion, CDC.

End-stage kidney disease (ESKD) (kidney failure requiring dialysis or transplantation) is a costly and disabling condition that often results in premature death (*1*). During 2019, Medicare fee-for-service expenditures for ESKD were $37.3 billion, accounting for approximately 7% of Medicare paid claims costs (*1*). Diabetes and hypertension remain the leading causes of ESKD, accounting for 47% and 29%, respectively, of patients who began ESKD treatment in 2019 (*1*). Compared with White persons, Black, Hispanic, and American Indian or Alaska Native persons are more likely to develop ESKD (*1*,*2*) and to have diagnosed diabetes (*3*). After declining for more than a decade, the incidence rate of ESKD with diabetes reported as the primary cause (ESKD from diabetes) has leveled off since 2010 (*1*,*4*). Further, after increasing for many years, the prevalence of diagnosed diabetes has also leveled off (*4*). Although these flattening trends in rates are important from a population perspective, the trend in the number of ESKD cases is important from a health systems resources perspective. Using United States Renal Data System (USRDS) 2000–2019 data, CDC examined trends in the number of incident and prevalent ESKD cases by demographic characteristics and by primary cause of ESKD. During 2000–2019, the number of incident ESKD cases increased by 41.8%, and the number of prevalent ESKD cases increased by 118.7%. Higher percentage changes in both incident and prevalent ESKD cases were among Asian, Hispanic, and Native Hawaiian or other Pacific Islander persons and among cases with hypertension or diabetes as the primary cause. Interventions to improve care and better manage ESKD risk factors among persons with diabetes and hypertension, along with increased use of therapeutic agents such as angiotensin-converting enzyme (ACE) inhibitors, angiotensin-receptor blockers (ARB), and sodium-glucose cotransporter 2 (SGLT2) inhibitors shown to have kidney-protective benefits (*5*,*6*) might slow the increase and eventually reverse the trend in incident ESKD cases.

USRDS collects, analyzes, and distributes ESKD clinical and claims data from the Centers for Medicare & Medicaid Services (CMS) Medical Evidence Report form (CMS 2728), which includes sociodemographic characteristics, the date patients were first treated for ESKD, and the primary cause of ESKD. The Medicare program covers 80% of the cost of ESKD treatment for beneficiaries in the United States regardless of age (*1*). Kidney care providers are required to complete the CMS 2728 form for each new patient with ESKD, regardless of Medicare eligibility status. Using USRDS 2000–2019 data, CDC examined the number of incident and prevalent ESKD cases in the United States each year during 2000–2019 by demographic characteristics (i.e., age, sex, and race/ethnicity) and by primary cause (i.e., diabetes, hypertension, or other cause). This activity was reviewed by CDC and was conducted consistent with applicable federal law and CDC policy.*

During 2000 and 2019, for both incident and prevalent ESKD cases, 34.9%–42.3% occurred among persons aged 45–64 years, 53.4%–-58.3% occurred among males, and 44.7%–55.2% occurred among White persons (Table). During 2000–2019, the number of incident ESKD cases increased 41.8%, from 92,660 to 131,422 (Table) (Figure 1), and the number of prevalent cases increased 118.7%, from 358,247 to 783,594 (Table) (Figure 2). Larger increases among incident cases occurred among Asian (149.5%), Native Hawaiian or other Pacific Islander (96.5%), and Hispanic (84.0%) persons (Table). Similarly, larger increases among prevalent cases were also observed among these populations. Smaller percentage increases in both incident and prevalent cases were observed among persons aged <45 years and among American Indian or Alaska Native persons. Although diabetes was the primary cause for a larger percentage of incident and prevalent ESKD cases, the largest increase in incident and prevalent cases was among patients with hypertension reported as the primary cause.

**TABLE:** Number of reported incident and prevalent cases of end-stage kidney disease, by selected characteristics — United States, 2000 and 2019*

Characteristic	Incident cases			Prevalent cases		
	2000	2019	Percentage change	2000	2019	Percentage change
	No. (%)^†^	No. (%)^†^		No. (%)^†^	No. (%)^†^	
**Total**	**92,660 (100.0)**	**131,422 (100.0)**	**41.8**	**358,247 (100.0)**	**783,594 (100.0)**	**118.7**
**Age group, yrs**						
<45	14,194 (15.3)	16,230 (12.3)	14.3	87,769 (24.5)	118,208 (15.1)	34.7
45–64	32,370 (34.9)	48,874 (37.2)	51.0	144,703 (40.4)	331,220 (42.3)	128.9
65–74	23,494 (25.4)	35,744 (27.2)	52.1	71,825 (20.0)	199,005 (25.4)	177.1
≥75	22,602 (24.4)	30,574 (23.3)	35.3	53,950 (15.1)	135,161 (17.2)	150.5
**Sex**						
Men	49,500 (53.4)	76,631 (58.3)	54.8	195,216 (54.5)	456,821 (58.3)	134.0
Women	43,160 (46.6)	54,791 (41.7)	26.9	163,031 (45.5)	326,773 (41.7)	100.4
**Race and Ethnicity**						
White	51,156 (55.2)	67,919 (51.7)	32.8	180,636 (50.4)	349,596 (44.7)	93.5
Black	25,917 (28.0)	33,700 (25.6)	30.0	116,376 (32.5)	234,399 (29.9)	101.4
Hispanic	11,297 (12.2)	20,790 (15.8)	84.0	42,129 (11.8)	140,961 (18.0)	234.6
Asian	2,507 (2.7)	6,256 (4.8)	149.5	11,839 (3.3)	41,393 (5.3)	249.6
American Indian or Alaska Native	1,041 (1.1)	1,299 (1.0)	24.8	4,538 (1.3)	7,949 (1.0)	75.2
Native Hawaiian or other Pacific Islander	742 (0.8)	1,458 (1.1)	96.5	2,729 (0.8)	9,296 (1.2)	240.6
**Primary cause**						
Diabetes	41,458 (44.7)	61,522 (46.8)	48.4	129,699 (36.2)	307,385 (39.2)	137.0
Hypertension	23,384 (25.2)	37,539 (28.6)	60.5	83,553 (23.3)	209,437 (26.7)	150.7
Other cause	27,818 (30.0)	32,361 (24.6)	16.3	144,995 (40.5)	266,772 (34.0)	84.0

* Data from United States Renal Data System, 2021 Annual Data Report, Reference Tables. https://adr.usrds.org/2021/reference-tables

^†^ Percentages might not sum to 100% because of rounding.

**FIGURE 1:**
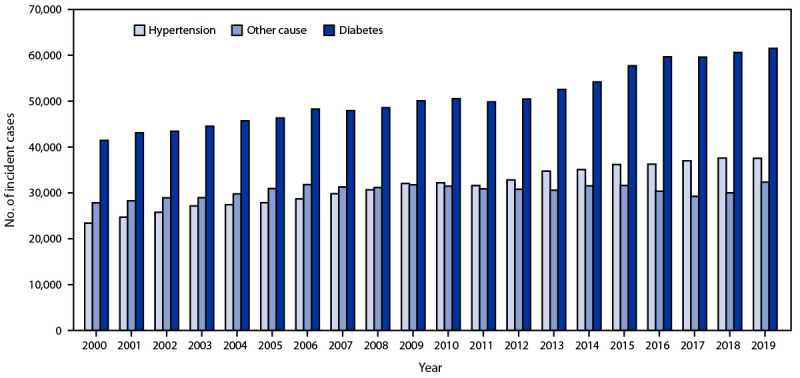
Number of reported incident cases of end-stage kidney disease, by primary cause — United States, 2000–2019* * Data from United States Renal Data System, 2021 Annual Data Report, Reference Tables. https://adr.usrds.org/2021/reference-tables

**FIGURE 2:**
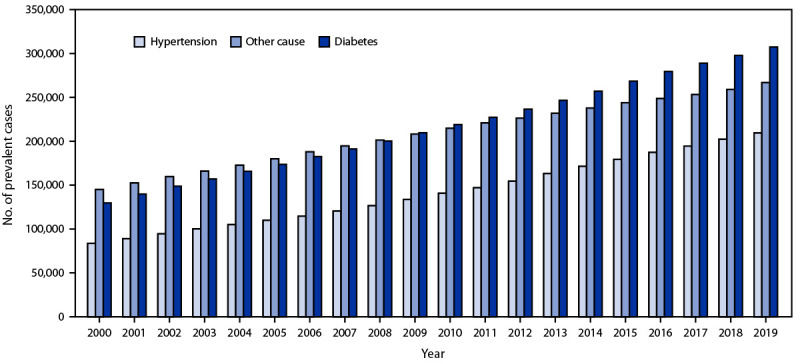
Number of reported prevalent cases of end-stage kidney disease, by primary cause — United States, 2000–2019* * Data from United States Renal Data System, 2021 Annual Data Report, Reference Tables. https://adr.usrds.org/2021/reference-tables

## Discussion

During 2000–2019, in the general U.S. population, the number of reported incident ESKD cases increased 41.8%, and the number of prevalent cases approximately doubled. Although persons aged 45–64 years, males, White persons, and persons with ESKD from diabetes accounted for the larger percentage of cases, Asian, Native Hawaiian or other Pacific Islander, Hispanic persons, and persons with ESKD from hypertension experienced the larger increase in cases. Compared with White persons, these racial/ethnic populations together with American Indian or Alaska Native and Black persons are disproportionately affected by ESKD (*1*). The continued increase in the number of ESKD cases will increase strain on the health care system and lead to higher costs. Effective management of diabetes and hypertension can help prevent ESKD and decrease the number of incident cases, thus alleviating the burden on the health care system and reducing costs.

Managing risk factors such as diabetes and high blood pressure and treatment with ACE inhibitors or ARBs have been shown to help prevent or delay the onset of ESKD from diabetes (*5*,*7*). In persons with diabetes, ACE inhibitors and ARBs lower blood pressure, reduce albuminuria, and slow the decline in kidney function (*5*). Other agents such as SGLT2 inhibitors have been shown to reduce the risks for cardiovascular disease and progression of chronic kidney disease in patients with type 2 diabetes, in addition to lowering blood glucose (*6*). However, the number of patients with newly treated ESKD from diabetes is likely to continue to increase with the increasing number of persons with diagnosed diabetes (*4*).

Compared with White persons, Black, Hispanic, and American Indian or Alaska Native persons are approximately two to three times as likely to develop ESKD (*1*,*2*). However, growth in incident and prevalent cases in the American Indian or Alaska Native population was slower than that in other populations. Population health and team-based approaches to diabetes care, including kidney disease testing and case management, implemented by the Indian Health Service, tribal and urban Indian health facilities, and supported by the Special Diabetes Program for Indians were associated with an estimated Medicare savings as high as $520.4 million in ESKD cases averted (*8*). This program might explain the lower percentage change in ESKD cases during 2000–2019. Expansion of these programs to other populations could reduce morbidity and save costs. In addition, interventions to promote and increase use of ACE inhibitors, ARBs, and SGLT2 inhibitors, along with improving care and better managing ESKD risk factors among persons with diabetes, might slow the increase and eventually reverse the trend in incident ESKD cases.

ESKD will continue to have a large impact on the U.S. health care system with population growth, aging, high prevalence of ESKD risk factors such as diabetes, better survival of the ESKD population, and improved transplant outcomes (*1*,*3*,*4*). Although the mortality rate in kidney transplant patients is three times lower compared with patients on dialysis (*1*), transplant recipients accounted for 3.0% of the incident and 29.6% of the prevalent ESKD cases in 2019. Further, annual transplant rates in this population declined somewhat during 2000–2019 (*1*). Several government agencies and nongovernmental organizations have implemented initiatives to increase access to kidney transplants and promote transplantation (*9*). In addition, CMS extended Medicare coverage of immunosuppressive drugs from 36 months to the lifetime of the kidney transplant recipient, preventing the return of transplant patients to dialysis. This extension of coverage is expected to save Medicare $400 million over 10 years (*10*). Whereas these factors collectively might result in the continued growth of the ESKD population, with better management of ESKD, patients can live a healthier life at a reduced cost to the health care system.

The findings in this report are subject to at least three limitations. First, data on ESKD treatment were based on reports to CMS; patients whose treatment was not reported to CMS (e.g., persons who refused treatment or died from ESKD before receiving treatment) were not included. Second, the primary cause of ESKD was obtained from the CMS Medical Evidence Report and was based on a physician’s assessment of the patient, which could be influenced by the physician’s awareness of a diabetes or hypertension diagnosis and not reflect the true cause of ESKD. Finally, differential classification of race or ethnicity in the CMS Medical Evidence Form could result in overcount or undercount of the actual number of ESKD cases in racial- or ethnic-specific groups.

One of the goals of the Advancing American Kidney Health Initiative of the U.S. Department of Health and Human Services is to reduce the number of Americans developing ESKD by 25% by 2030 (*9*). Effective management of diabetes and hypertension, including kidney disease testing and management as part of diabetes care in at-risk populations, can help prevent ESKD. Monitoring trends and racial or ethnic disparity gaps in ESKD, and tracking other factors such as kidney disease awareness, pre-ESKD care, and risk factor (e.g., diabetes or hypertension) control and prevention, will be very important to evaluate the success of these interventions. Continued efforts to address ESKD risk factors to prevent or delay ESKD onset could stabilize or reverse the increase in the number of persons living with ESKD.

**Summary**

**What is already known about this topic?**

End-stage kidney disease (ESKD) (kidney failure requiring dialysis or transplantation) is a disabling condition that often results in premature death. ESKD is costly, accounting for $37.3 billion of Medicare expenditures during 2019.

**What is added by this report?**

During 2000–2019, the number of ESKD cases reported in the United States increased 41.8%; the number of prevalent cases approximately doubled. Higher percentage changes in incident and prevalent ESKD cases were attributable to primary causes related to diabetes and hypertension.

**What are the implications for public health practice?**

Effective management of diabetes and hypertension can help prevent ESKD and decrease the number of incident cases, thus reducing costs and alleviating the impact on the health care system.

## Footnotes

* 45 C.F.R. part 46.102(l)(2); 21 C.F.R. part 56; 42 U.S.C. Sect. 241(d); 5 U.S.C. Sect. 552a; 44 U.S.C. Sect. 3501 et seq.
